# Longitudinal changes in physical activity and sedentary time in adults around retirement age: what is the moderating role of retirement status, gender and educational level?

**DOI:** 10.1186/s12889-016-3792-4

**Published:** 2016-10-28

**Authors:** Delfien Van Dyck, Greet Cardon, Ilse De Bourdeaudhuij

**Affiliations:** 1 Research Foundation Flanders (FWO), Egmontstraat 5, 1000 Brussels, Belgium; 2 Ghent University, Department of Movement and Sport Sciences, Watersportlaan 2, 9000 Ghent, Belgium

**Keywords:** Exercise, TV viewing, Computer use, Moderators, Longitudinal study, Socio-demographic factors

## Abstract

**Background:**

The start of retirement is an important stage in an (older) adult’s life and can affect physical activity (PA) and/or sedentary behaviors, making it an ideal period to implement health interventions. To identify the most optimal timing of such interventions it is important to determine how PA and sedentary behaviors change not only when making the transition to retirement, but also during the first years of retirement. The main study aim was to examine whether PA and sedentary behaviors change differently in retiring adults compared with recently retired adults. A second aim was to examine potential moderating effects of gender and educational level.

**Methods:**

A longitudinal study was conducted in Ghent, Belgium. Baseline measurements took place in 2012–2013 and follow-up data were collected 2 years later. In total, 446 adults provided complete data at both time points. Of the participants 105 adults were not retired at baseline but retired between baseline and follow-up (i.e. retiring) and 341 were already retired at baseline (i.e. recently retired). All participants completed a questionnaire on PA, sedentary behaviors, socio-demographic factors and physical functioning. Repeated measures MANOVAs were conducted in SPSS 22.0. to analyze the data.

**Results:**

Leisure-time cycling increased over time in retiring adults, but decreased in recently retired adults (*p* < 0.01). (Voluntary) work-related walking and moderate-to-vigorous PA decreased strongly in retiring adults, while slight increases were found in recently retired adults (*p* < 0.001 and *p* < 0.01). Passive transport decreased more strongly in recently retired than in retiring adults (*p* < 0.05), and computer use increased more in retiring adults than in the recently retired group (*p* < 0.001). Low-educated recently retired adults had the strongest decrease in walking for transport (*p* < 0.05) and strongest increase in TV viewing time (*p* < 0.01) and computer use (*p* < 0.10). For gender, almost no moderating effects were found.

**Conclusions:**

Future interventions should focus on PA and/or specific sedentary behaviors in retiring adults, but should definitely include long-term follow-up, as recently retired adults seem to be prone to lapse into an unhealthy lifestyle. Specific attention should be paid to low-educated adults as they are particularly susceptible to a decrease in PA and increased TV viewing time and computer use.

**Electronic supplementary material:**

The online version of this article (doi:10.1186/s12889-016-3792-4) contains supplementary material, which is available to authorized users.

## Background

Both in developed and developing countries, life expectancy has increased steadily over the last decades [1]. It is estimated that worldwide, the population aged 60 years and older will double from about 11 % in 2013 to 21 % in 2050 [2]. A major challenge associated with this increase in life expectancy is the increase in health care costs due to age-related chronic diseases (e.g. cardio-vascular diseases, type 2 diabetes) [3]. To prevent or delay the development of such chronic diseases, a healthy lifestyle with sufficient physical activity (PA) and limited sedentary time is necessary [4, 5]. Nonetheless, with increasing age PA typically declines and sedentary behaviors (e.g. TV viewing, reading) increase [6, 7].

The transition to retirement is an important turning point in an adult’s life. Retirement can be defined as ‘a permanent and complete withdrawal from the labor force’, and goes together with changes in social networks, income and time flexibility [8]. All these changes can influence PA and sedentary behaviors. Several longitudinal studies examined how the transition to retirement affects PA and/or sedentary behaviors by comparing retiring adults (i.e. adults who are not retired at baseline and retire between baseline and follow-up) with adults who continued to work [9–12]. These longitudinal studies found that the transition to retirement was associated with an increase in leisure-time PA. Nonetheless, compared to adults who continued to work, retiring adults had a stronger decrease in total PA, mainly due to decreases in active transport and work-related PA, and a stronger increase in TV viewing time. Based on these findings, it has been suggested that health promotion interventions aiming to increase PA around retirement should target individuals who are planning to retire in the near future.

However, one should not only compare longitudinal changes in PA and sedentary behaviors between retiring adults and working adults, but also between retiring adults and adults who have been retired for a longer period. In that way, it can be clarified if PA and sedentary behaviors change differently during the transition to retirement compared to after retirement and the most optimal timing of health promotion interventions targeted to adults around the retirement age, can be determined. It might be the case that specific types of PA like leisure-time and household PA increase in adults who make the transition to retirement, but decrease in recently retired adults who are accustomed to their new status of being retired. If so, it may be more effective to target adults who are recently retired instead of adults who will retire in the near future. The only previous longitudinal study that compared retiring adults with retired adults (i.e. retired at baseline and follow-up measurements) confirmed this and reported that after controlling for age, leisure-time PA and household PA increased in retiring adults, while a decrease in leisure-time PA and no change in household PA were identified in retired adults [13]. Nonetheless, for TV viewing time and computer use opposite findings were reported (stronger increase in retiring than in retired adults) [13], so more research, preferably including a broader range of sedentary behaviors, is needed in order to formulate firm recommendations about the timing (i.e. before retirement or some years after retirement) and specific content (i.e. which behaviors to focus on) of interventions.

In addition to examining potential differences in longitudinal changes in PA and sedentary behaviors between retiring and recently retired adults, it is also important to take into account the potential moderating effects of socio-economic status (SES) and gender. Previous studies found a stronger decline in overall PA among retiring adults from lower social classes than those from higher social classes [10, 14]. This is probably due to the fact that adults from lower social classes rather engage in manual jobs and consequently have higher levels of work-related PA than adults from higher social classes during their working career. The greater loss of work-related (and transport-related) PA is probably not sufficiently compensated by an increase in leisure-time or other types of PA [10, 14]. This was also confirmed in a qualitative study showing that adults with a physically demanding job reported that retirement is a period of well-deserved rest after an active career [15]. Moreover, TV viewing increased more strongly among retiring adults from lower social classes [14]. Concerning gender, a systematic review reported that the increase in leisure-time PA during early retirement is slightly higher in men than in women [8]. No information about the potential moderating effects of gender on longitudinal changes in sedentary behaviors in recently retired and retiring adults is available yet.

The current longitudinal study has two aims. First, we will examine whether a broad range of physical activities and sedentary behaviors change differently in retiring adults (i.e. adults who retire between baseline and follow-up) compared with recently retired adults (i.e. adults who are retired for at least 6 months and maximum 5 years at baseline). By doing so, we aim to formulate specific guidelines on the most optimal timing and content of health promotion interventions in adults around the retirement age. Secondly, we will examine whether these differences in changes in PA and sedentary behaviors between retiring and recently retired adults depend on gender and educational level.

## Methods

The present study was conducted in Ghent (250,000 inhabitants, 156.18 square km (60.3 mile^2^), 1601 inhabitants/square km), Flanders, Belgium. Baseline data were collected in two waves, a first wave in December 2012 and a second wave in May 2013. Follow-up data were similarly collected in two waves, 2 years after baseline data collection (December 2014, May 2015).

### Procedures and participants

At baseline, individuals who retired recently (>6 months, <5 years of retirement) and individuals who planned to retire within the next 18 months were targeted. In Flanders, the formal retirement age of the current workforce over 50 years of age varies between 58 and 65 years [16], but official records with information on retirement status are not publicly available. Consequently, in order to recruit a sufficient number of recently retired adults and of adults who were planning to retire within the next 18 months, the Public Service of Ghent selected a random sample of 7500 58–65 year old adults from the municipal register. At baseline, all these adults received an invitation letter with information on the study (2500 adults in December 2012, 5000 adults in May 2013). After 2 weeks, a reminder was sent. Only adults who planned to retire within the next 18 months, and those who had been retired for more than 6 months but less than 5 years could participate in the study. Recently retired adults needed to be full-time retired from their main occupation, but engaging in voluntary work was allowed. Furthermore, as PA was one of the outcome variables in this study, participants had to be able to walk 100 m without assistance in order to be eligible for the study. Adults who were willing to participate in the study and met the inclusion criteria, were asked to confirm their participation by phone or email. These adults received a postal questionnaire (with a pre-stamped envelope to return the questionnaire) including questions on socio-demographic characteristics, PA and sedentary time, and physical and mental health. In total, 597 adults (455 recently retired, 142 planning to retire) returned a complete questionnaire. Because it is unknown how many of the 7500 addressed adults were truly eligible to participate in the study, it is not possible to calculate the response rate.

After 2 years (December 2014 and May 2015) these 597 adults received the same postal questionnaire again (follow-up measurements). In total, 463 adults (77.6 %) returned a complete questionnaire at follow-up. Of these 463 participants, five were not yet retired, three did not report the month/year of retirement, and nine participants had not been working before they officially retired (seven housewives and two disabled persons). Consequently, the final sample used in the analyses consisted of 446 participants (341 recently retired adults (i.e. already retired at baseline) and 105 adults who retired between baseline and follow-up). The study protocol was approved by the ethics committee of the Ghent University Hospital (B670201215326). Written informed consent was obtained from all participants.

### Measures

#### Physical activity and sedentary time

Self-reported PA was measured with the International Physical Activity Questionnaire (IPAQ; long past 7 days version). PA assessed by the IPAQ showed good reliability (intra-class correlations range from 0.46 to 0.96) and fair-to-moderate criterion validity compared against accelerometers (median ρ 0.30) in a 12-country study [17]. Frequency (number of days) and duration (minutes/day) of PA in different domains were queried. Based on this information, separate estimates of weekly minutes of cycling for transport, walking for transport, household-related moderate PA, moderate-to-vigorous PA (MVPA) during gardening, (voluntary) work-related walking and MVPA, leisure-time cycling, leisure-time walking and leisure-time MVPA were calculated.

Self-reported minutes/week of passive transport, TV viewing, computer use, sitting during hobbies, household chores and sitting during meals were assessed using a translated (Flemish) version of the leisure-time sedentary behavior questionnaire developed by Salmon and colleagues [18]. The English-language version of the questionnaire has fair to excellent reliability (intra-class range from 0.56 to 0.82). Concurrent validity, assessed against a 3-day behavioral log was fair-to-moderate, with rho’s ranging from 0.20 to 0.60 [18].

#### Socio-demographic information and physical functioning

Self-reported socio-demographics included gender, age, weight, height, current marital status (alone, married, living together, widowed, divorced), and educational level (primary, secondary, tertiary education). BMI was calculated by dividing the weight (kg) by the height (m) squared. For the analyses, educational level was dichotomized into high education (i.e. tertiary education) versus low education (i.e. primary and secondary education). Self-reported physical functioning was assessed with the physical functioning subscale of the Short Form 36 item Survey (SF-36) [19]. This subscale comprises ten activities (e.g. moderate activities, climb several flights, bend or kneel) and participants were asked to report on a three-point scale whether or not they were restricted by their physical health to perform these activities (severely limited; somewhat limited; not limited). Based on these answers, an overall score of physical functioning was calculated, ranging from 0 to 100 with a higher score representing better physical functioning. This calculation was based on the standard protocol to process the SF-36 [19].

### Statistical analyses

**Fig. 1:**
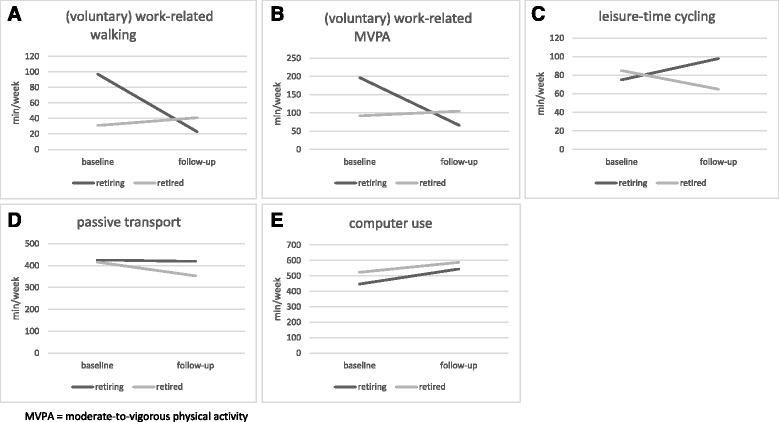
(Panels **a**-**e**) Changes in physical activities and sedentary behaviors: differences between retiring and retired adults

All data were analyzed using SPSS 22.0. Baseline demographic characteristics were compared between recently retired and non-retired participants using independent samples t-tests and chi-square tests. To examine whether longitudinal changes in PA and sedentary behaviors differed between retiring and recently retired adults, and to examine whether this effect was dependent on (1) educational level and (2) gender, repeated measures MANOVAs with time (baseline/follow-up) as within-subjects factor and retirement status (recently retired/retiring) as between-subjects factor were conducted. Educational level and gender were alternately entered as an additional between-subjects factor. In total, four MANOVA models were constructed: two models for self-reported PA (nine measures: walking and cycling for transport, household-related moderate PA, MVPA during gardening, (voluntary) work-related walking and MVPA, leisure-time walking, cycling and MVPA), of which one model with educational level and one with gender as additional between-subjects factor; and two (again one with educational level and one with gender as additional between-subjects factor) for self-reported sedentary time (six measures: passive transport, TV viewing, computer use, sitting during hobbies, during household chores and sitting during meals). Because all PA and sedentary time variables were positively skewed, logarithmic transformations (log10) were applied to improve normality [20]. The repeated measures MANOVAs were conducted using these transformed variables, but for ease of interpretation, raw descriptive statistics are reported in Table 1. Time by retirement status interactions were used to test if the changes in PA and sedentary behaviors differed between retiring and recently retired adults. Significant interactions are presented graphically in Fig. 1. Time by retirement by educational level (respectively gender) interactions were used to test if the effect of retirement status differed according to educational level/gender. In order to report complete results, also time effects are reported in Table 2, but these effects were not interpreted in further detail. Statistical significance was set at *p* < 0.05 but because of the relatively small sample size, marginally significant results (*p* < 0.10) were also reported.

**Table 1:** Physical activity and sedentary behaviors at baseline and two-year follow-up: descriptive statistics

	Total group Mean (SD) *n* = 446	Retired Mean (SD) *n* = 341	Retiring Mean (SD) *n* = 105	High education		Low education		Men		Women	
				Retired Mean (SD) *n* = 154	Retiring Mean (SD) *n* = 56	Retired Mean (SD) *n* = 185	Retiring Mean (SD) *n* = 49	Retired Mean (SD) *n* = 173	Retiring Mean (SD) *n* = 61	Retired Mean (SD) *n* = 167	Retiring Mean (SD) *n* = 44
Physical Activity (min/week)											
Walking transport											
Baseline	181 (228)	179 (234)	187 (209)	148 (201)	164 (177)	210 (260)	207 (234)	155 (230)	176 (198)	205 (236)	203 (226)
Two-year follow-up	171 (217)	167 (211)	184 (238)	168 (208)	158 (211)	162 (210)	208 (259)	151 (201)	151 (213)	184 (220)	230 (264)
Cycling transport											
Baseline	70 (134)	65 (123)	86 (165)	63 (109)	65 (125)	69 (139)	104 (193)	68 (124)	81 (161)	60 (121)	92 (173)
Two-year follow-up	71 (139)	66 (123)	87 (180)	62 (112)	76 (151)	70 (136)	97 (204)	69 (123)	60 (100)	184 (220)	124 (248)
MVPA garden											
Baseline	180 (332)	204 (366)	105 (162)	222 (373)	140 (191)	183 (359)	73 (124)	298 (453)	131 (183)	107 (210)	68 (119)
Two-year follow-up	154 (278)	160 (291)	133 (234)	153 (254)	158 (231)	171 (331)	111 (236)	204 (334)	165 (248)	112 (227)	89 (207)
Moderate PA household											
Baseline	259 (284)	277 (289)	199 (256)	246 (279)	133 (189)	308 (295)	256 (293)	206 (270)	146 (219)	352 (292)	273 (287)
Two-year follow-up	248 (282)	256 (283)	223 (279)	245 (278)	172 (249)	266 (286)	269 (299)	192 (256)	154 (234)	322 (295)	320 (310)
Walking (voluntary) work											
Baseline	47 (136)	31 (106)	97 (197)	25 (96)	67 (173)	39 (119)	123 (215)	40 (120)	91 (200)	23 (90)	104 (196)
Two-year follow-up	37 (117)	41 (121)	23 (100)	38 (116)	29 (112)	45 (128)	18 (89)	41 (120)	20 (74)	41 (123)	28 (128)
MVPA (voluntary) work											
Baseline	117 (300)	92 (257)	197 (401)	66 (213)	114 (321)	121 (299)	270 (450)	115 (292)	194 (408)	67 (214)	202 (395)
Two-year follow-up	96 (257)	105 (267)	66 (218)	89 (238)	40 (177)	126 (300)	88 (248)	122 (290)	72 (202)	88 (242)	57 (241)
Walking leisure-time											
Baseline	146 (224)	146 (222)	146 (230)	149 (227)	128 (218)	136 (210)	162 (241)	130 (214)	143 (226)	162 (231)	151 (238)
Two-year follow-up	140 (215)	131 (203)	171 (248)	131 (192)	156 (225)	127 (210)	184 (268)	115 (190)	144 (229)	149 (215)	207 (272)
Cycling leisure-time											
Baseline	82 (181)	85 (181)	75 (180)	85 (182)	84 (182)	85 (182)	67 (179)	115 (214)	104 (221)	53 (133)	35 (86)
Two-year follow-up	73 (165)	65 (159)	98 (182)	62 (145)	129 (211)	69 (174)	72 (149)	80 (170)	133 (211)	50 (145)	51 (119)
MVPA leisure-time											
Baseline	105 (234)	102 (221)	113 (271)	115 (230)	90 (194)	87 (211)	134 (324)	109 (235)	139 (322)	96 (207)	78 (173)
Two-year follow-up	87 (201)	86 (193)	91 (225)	93 (195)	99 (202)	79 (193)	84 (246)	102 (222)	107 (266)	67 (154)	69 (154)
Sedentary behaviors (min/week)											
Passive transport											
Baseline	418 (437)	416 (436)	426 (445)	377 (374)	456 (473)	445 (470)	399 (422)	461 (451)	517 (491)	370 (417)	299 (339)
Two-year follow-up	368 (394)	353 (366)	419 (469)	346 (374)	424 (472)	361 (359)	415 (470)	399 (394)	454 (493)	299 (323)	371 (433)
TV viewing											
Baseline	898 (621)	921 (619)	824 (626)	741 (530)	678 (571)	1137 (649)	952 (648)	892 (641)	809 (708)	947 (595)	845 (497)
Two-year follow-up	1048 (644)	1055 (656)	1025 (607)	910 (633)	861 (472)	1236 (639)	1169 (676)	1016 (630)	966 (673)	1092 (683)	1107 (495)
Computer use											
Baseline	505 (526)	523 (499)	447 (606)	531 (472)	535 (747)	517 (531)	371 (441)	630 (564)	489 (631)	413 (395)	390 (572)
Two-year follow-up	578 (564)	588 (591)	545 (464)	579 (546)	556 (351)	578 (605)	536 (548)	669 (571)	562 (526)	504 (604)	522 (366)
Sitting during hobby’s											
Baseline	687 (680)	702 (680)	640 (679)	755 (644)	627 (690)	640 (719)	652 (676)	672 (707)	576 (709)	736 (653)	729 (633)
Two-year follow-up	730 (717)	731 (748)	726 (608)	756 (731)	662 (515)	682 (742)	782 (680)	671 (770)	682 (599)	798 (721)	788 (623)
Sitting household chores											
Baseline	114 (307)	132 (337)	57 (173)	127 (234)	52 (140)	138 (431)	62 (199)	118 (245)	28 (82)	145 (411)	97 (246)
Two-year follow-up	124 (323)	137 (353)	83 (195)	132 (318)	71 (168)	146 (393)	94 (217)	130 (327)	78 (192)	145 (379)	90 (200)
Sitting during meals											
Baseline	667 (320)	673 (316)	648 (331)	645 (267)	634 (335)	702 (357)	659 (330)	622 (287)	641 (358)	727 (336)	656 (294)
Two-year follow-up	659 (338)	666 (349)	636 (299)	657 (326)	636 (293)	670 (368)	636 (307)	644 (337)	597 (294)	690 (361)	691 (301)

*SD* standard deviation; *MVPA* moderate-to-vigorous physical activity; *PA* physical activity

**Table 2:** Two-year changes in physical activity and sedentary behaviors by retirement status, educational level and gender

	Time F	Time × Retirement status F	Time × Retirement status × educational level F	Time × Retirement status × gender F
Physical Activity (min/week)				
Walking transport	4.62*	2.05	4.07*	2.66
Cycling transport	0.26	0.56	1.36	0.99
MVPA garden	2.47	0.40	0.06	3.07^£^
Moderate PA household	0.16	2.63	1.03	1.18
Walking work	2.47	18.32***	3.32^£^	2.87^£^
MVPA work	0.77	8.01**	2.61	3.58^£^
Walking leisure-time	0.31	0.32	0.08	1.37
Cycling leisure-time	0.51	7.99**	0.58	0.19
MVPA leisure-time	1.50	0.75	0.67	0.42
Sedentary behaviors (min/week)				
Passive transport	0.24	4.48*	0.29	10.18**
TV viewing	0.56	0.03	7.52**	0.03
Computer use	2.54	23.28***	2.76^£^	3.71^£^
Sitting during hobby’s	0.35	0.36	0.25	0.01
Sitting household chores	0.44	1.02	0.92	0.70
Sitting during meals	5.23*	0.50	1.42	0.97

^£^ *p* < 0.10; *p < 0.05; **p < 0.01; ***p < 0.001

## Results

### Characteristics of the study sample

Mean age of the participants at baseline was 62.4 (SD 2.2) years, mean BMI was 25.8 (SD 4.3) kg/m^2^ and mean level of physical functioning was 86.1 (SD 15.1). In total, 52.6 % of the sample was male, 52.7 % had a college/university degree and 74.9 % lived with a partner. For the group of adults who were already retired at baseline (i.e. recently retired group), the average duration of retirement was 26 months (SD 18 months). Chi-square tests and independent sample t-tests showed that there were no significant baseline differences in gender, marital status BMI, educational level, and in physical functioning between the participants who were recently retired at baseline and those who were not yet retired. Recently retired adults were older (mean age 62.8, SD 2.0) than retiring adults (mean age 61.3, SD 2.6) at baseline (*p* < 0.001). Consequently, age was included as a covariate in the repeated measures MANOVAs.

### Longitudinal changes in PA and sedentary time: differences between retiring and recently retired adults

Descriptive statistics on PA and sedentary behaviors are presented in Table 1. Results of the Repeated Measures MANOVA analyses can be found in Table 2. Regarding PA, significant time by retirement status interactions were found for (voluntary) work-related walking (*p* < 0.001), (voluntary) work-related MVPA (*p* < 0.01) and leisure-time cycling (*p* < 0.01) (Fig. 1 – panels a to c). Both (voluntary) work-related walking and (voluntary) work-related MVPA decreased strongly adults who retired between baseline and follow-up (on average −74 min/week and −131 min/week respectively) while small increases were found in adults who were recently retired at baseline (on average +10 min/week and +13 min/week respectively). Mean minutes/week of leisure-time cycling increased in retiring participants (+22 min/week), while a decrease was found in participants who were recently retired at baseline (−20 min/week). For transport-related walking and cycling, MVPA during gardening, household-related moderate PA, leisure-time walking and leisure-time MVPA, no significant time by retirement status interactions were found.

Regarding sedentary behaviors, significant interactions were found for passive transport (*p* < 0.05) and computer use (*p* < 0.001) (Fig. 1 – panels d and e). Passive transport decreased less strongly in retiring adults (-7 min/week) than in recently retired adults (−63 min/week). Computer use increased stronger in retiring participants (+98 min/week) than in participants who were already retired at baseline (+65 min/week). For the other sedentary behaviors, no significant time by retirement status interactions were identified.

### Differences in longitudinal changes in PA and sedentary time between retiring and recently retired adults: moderating effects of educational level

For PA, (marginally) significant time by retirement status by educational level (three-way) interactions were found for walking for transport (*p* < 0.05) and (voluntary) work-related walking (*p* < 0.10). The interaction effect identified for walking for transport showed that in low-educated participants, only recently retired adults showed a decrease (−48 min/week versus +1 min/week for retiring adults), while in high-educated participants, retiring adults slightly decreased their transport-related walking (−6 min/week) and recently retired adults increased their transport-related walking (+20 min/week). For (voluntary) work-related walking, results showed that in both high- and low-educated adults, participants who were retired at baseline slightly increased their (voluntary) work-related walking (+13 min/week and +6 min/week respectively) and retiring participants strongly decreased. However, the decrease in retiring participants was larger in low-educated adults (-108 min/week versus −38 min/week in high-educated adults). For the other PA behaviors, no significant three-way interactions were found.

For the sedentary behaviors, (marginally) significant three-way interactions were identified for TV viewing (*p* < 0.01) and computer use (*p* < 0.10). For TV viewing, the results showed that in high-educated adults, a similar increase in TV viewing time was found for recently retired (+169 min/week) and retiring (+183 min/week) adults. However, in low-educated adults, retiring adults increased their TV viewing time much stronger (+217 min/week) than recently retired adults (+99 min/week). A similar interaction effect was found for computer use: in low-educated adults, retiring adults increased their computer use much stronger (+165 min/week) than adults who were already retired at baseline (+61 min/week), while in high-educated adults, increases were relatively similar for retiring (+21 min/week) and recently retired adults (+48 min/week). For the other sedentary behaviors, no significant time by retirement status by educational level interactions were found.

### Differences in longitudinal changes in PA and sedentary behaviors between retiring and recently retired adults: moderating effects of gender

For PA, marginally significant time by retirement status by gender (three-way) interactions were found for MVPA during gardening, (voluntary) work-related walking and (voluntary) work-related MVPA (all *p* < 0.10). The interaction effect identified for MVPA during gardening showed that MVPA only decreased in men who were already retired at baseline (−94 min/week), while increases were found in retiring men (+34 min/week), retiring women (+21 min/week) and recently retired women (+5 min/week). For (voluntary) work-related walking, results showed that in both men and women, retiring adults strongly decreased (respectively −71 min/week and −76 min/week), while recently retired women increased their (voluntary) work-related walking (+18 min/week) and retired men showed almost no change (+1 min/week). Similarly, for (voluntary) work-related MVPA, both retiring men and women showed strong decreases (−122 min/week and −145 min/week), while recently retired women increased their (voluntary) work-related MVPA more strongly (+21 min/week) than recently retired men (+7 min/week). For the other PA behaviors, no significant three-way interactions were found.

For the sedentary behaviors, (marginally) significant three-way interactions were identified for passive transport (*p* < 0.01) and computer use (*p* < 0.10). Both recently retired men and women showed a similar decrease in passive transport (−62 min/week and −71 min/week). However, retiring men showed a decrease in passive transport (-63 min/week), while retiring women showed an increase (+72 min/week). All groups increased their computer use, but the increase was strongest in retiring women (+131 min/week), followed by recently retired women (+91 min/week) and retiring men (+73 min/week), and less pronounced in recently retired men (+39 min/week). For the other sedentary behaviors, no significant three-way interactions were found.

## Discussion

The first aim of this study was to examine whether 2-year changes in specific physical activities and sedentary behaviors differed between retiring adults (i.e. adults who retired between baseline and follow-up) and recently retired adults (i.e. adults who were retired for at least 6 months and maximum 5 years at baseline). Our findings showed that leisure-time cycling increased in retiring adults, but decreased in recently retired adults. Furthermore, (voluntary) work-related walking and MVPA decreased strongly in retiring adults, while a slight increase over time was found in the recently retired group. Regarding sedentary behaviors, passive transport decreased more strongly in recently retired adults than in retiring adults, and computer use increased more strongly in retiring adults compared with recently retired adults.

These findings are partly in line with previous results of Menai and colleagues [13], who also found that retiring adults’ leisure-time PA increased, while retired adults’ leisure-time PA decreased. However, they also found an increase in household PA in retiring adults, while our findings could not confirm this. Concerning TV viewing and computer use, Menai and colleagues [13] concluded that the increases were larger in retiring adults than in retired adults. No other types of sedentary behavior were included in their study, nor was (voluntary) work-related PA. The strong decrease in (voluntary) work-related walking and MVPA found in retiring adults in the present study is very logical. The fact that voluntary work-related PA increases in recently retired adults is promising as it means that some of them fill up their free time with active voluntary work. Furthermore, our results, as well as the previous findings of Menai and colleagues [13] confirm the hypothesis that adults increase specific types of PA (leisure-time cycling) at the start of retirement, probably because the transition to retirement induces reduced time constraints and because many adults perceive the start of retirement as a moment to take up healthy habits and to replace their working-day routine with new routines [21, 22]. Nonetheless, a decrease in cycling was identified in adults who were already retired at baseline. Based on these findings, it seems like for specific PA types, adults tend to lapse into old habits once they are accustomed to retirement. Previous qualitative research showed that individuals subdivide ‘retirement’ in different temporary phases, each with distinct PA patterns [23]. So, later phases of retirement may imply decreased levels of PA. However, in order to firmly confirm this suggestion, changes in PA patterns of retiring adults should be followed up during a longer period (e.g. from before retirement until 5 or more years after retirement).

Concerning sedentary behaviors, our findings are not very straightforward. A positive change (i.e. decrease) was identified for passive transport, while computer use increased, and for both behaviors changes were more pronounced in retiring adults. Most previous studies only included TV viewing time when examining changes in sedentary behavior [10, 11], but our results confirm that computer use is becoming highly prevalent in older adults, and should certainly be included as a sedentary behavior to target in future studies. The identified decrease in passive transport in retiring adults is very positive, not only for health, but also for the environment (e.g. pollution), and is probably mainly related to quitting work. The continued decrease in passive transport in recently retired adults might have less positive consequences too: old age is often associated with increased isolation and decreased mobility, so it might be the case that retired adults are more ‘tied’ to their home [24]. In future research the reasons for this decrease in passive transport should be examined in more detail. Overall, our results confirm the need to include several sedentary behaviors in future studies, instead of focusing only on TV viewing time or overall sedentary time.

The second aim was to examine if educational level and gender moderated the effects of retirement status (retiring versus recently retired) on the 2-year changes in PA and sedentary behaviors. Concerning the moderating effects of educational level, our findings showed that low-educated recently retired adults had the strongest decrease in walking for transport, while low-educated retiring adults’ work-related walking decreased most strongly. Previous studies showed that low-educated adults (who often have a physically demanding job, which was also the case in our study [results not shown]) have stronger decreases in PA during the transition to retirement than high-educated adults, on the one hand because they usually do more work-related PA before retirement, and on the other hand because they often believe retirement is a period of well-deserved rest [10, 14]. Our findings on transport-related walking confirm that also after retirement, low-educated adults are an important at-risk group for having low PA levels. Furthermore, TV viewing time and computer use increased most strongly in low-educated retiring adults, which confirms the susceptibility for low-educated adults to lapse into an unhealthy lifestyle around and after the transition to retirement. Nonetheless, no moderating effects of educational level were found for most other PA and sedentary behaviors, so it seems that future interventions may be most effective when both high- and low-educated adults are targeted.

Regarding the moderating effects of gender, our results showed that work-related walking and MVPA decreased in recently retired men, while increases were identified in recently retired women; MVPA during gardening decreased strongly in recently retired men, and increased in retiring men, retiring women and retired women. However, retiring women showed strong increases in passive transport and computer use while retiring men’s passive transport decreased and computer use increased moderately. So, these findings demonstrate that retiring women are inclined to increase MVPA during gardening, but also their passive transportation and computer use, more than retiring men. Almost no previous studies examined gender differences, but those that did found post-retirement increases in PA to be slightly higher in men than in women [8], which conflicts with our results.

Previous studies carefully suggested that interventions aiming to increase PA around the age of retirement, should target individuals who retired recently or who are planning to retire in the near future [9–12]. However, those studies only compared retiring adults with adults who continued to work, and did not include recently retired individuals. So, based on our study, these recommendations can be somewhat adapted and fine-tuned. Our findings suggest that interventions should focus not only on retiring, whose overall PA tends to decrease because the decline of work-related PA is not sufficiently compensated by increases in other domains. Future research should also pay attention to long-term follow-up as the changes in behavior seem to be dependent on the specific stage of retirement adults are facing. Furthermore, interventions should also focus on sedentary behaviors, with specific attention for sedentary time during computer use in both retiring and recently retired adults. Low-educated retiring and recently retired adults seem to be particular at-risk groups for insufficient transport- and voluntary work-related walking and high levels of TV viewing and computer use, so they should receive specific attention in interventions. Nonetheless, since no moderating effects of educational level were found for the other behaviors included in the study, it can be recommended to target both high- and low-educated adults in interventions. No straightforward results were identified regarding the moderating role of gender, so currently, tailoring of interventions towards retiring/recently retired men or women cannot be recommended.

Strengths of this study firstly include that a broad range of PA and sedentary behaviors were examined. Previous studies mainly focused on changes in leisure-time PA and TV viewing time, or overall PA and sedentary time. Secondly, this study had a strong (longitudinal) design. Furthermore, some limitations should be acknowledged. First, a relatively small sample of retiring adults (*n* = 105) participated, which limits the power of our analyses and the generalizability of our findings. The three-way interaction analyses were particularly underpowered with approximately 50 participants in each group of retiring adults (i.e. retiring men, retiring women, retiring low-educated adults and retiring high-educated adults). Second, follow-up time was limited to 2 years. Future studies on health behaviors over the retirement transition should include more participants and should follow the same cohort of participants over a longer period (e.g. from before retirement to 5 years post retirement). Third, selection bias was probably present as only 446 of the 7500 adults who received an invitation letter for the study, completed both baseline and follow-up measurements. It may thus be the case that mainly motivated and active adults responded to the invitation letter. Fourth, only self-reported PA and sedentary behaviors were included. As the use of questionnaires is subject to several biases (e.g. recall bias, social desirability), it is recommended to combine this with objective assessments of PA and sedentary time in future studies.

## Conclusion

In conclusion, this study showed that leisure-time cycling tends to increase in retiring adults, but decreases in recently retired adults. Furthermore, as expected, retiring adults showed a strong decrease in (voluntary) work-related PA, while small increases in voluntary work-related PA were found in recently retired adults. Retiring adults, and to a lesser extent recently retired adults are also at risk of increasing their sedentary time due to increased computer use. Future interventions should focus on PA and/or specific sedentary behaviors in retiring adults, and should definitely include long-term follow-up as behavior changes seem to be different across diverse phases of retirement. Finally, specific attention should be paid to low-educated retiring and recently retired adults as they are particularly susceptible to a decrease in PA and increased TV viewing time and computer use.

## Additional file

Additional file 1: Data file used for statistical analyses reported in this paper. (CSV 769 kb)

## Abbreviations

BMI: Body mass index
PA: Physical activity

## References

[1] World Health Organization. Health topics. Ageing and life course. Geneva, Switzerland, 2006. Available at: http://www.who.int/ageing/en. Accessed 18 Apr 2016.

[2] United Nations, Department of Economic and Social Affairs PD (2013). World Population Ageing 2013.

[3] Organization for Economic Cooperation and Development (2006). Study projects growing pressure on public health spending over and above effects of ageing society.

[4] Hamilton MT, Healy GN, Dunstan DW, Zderic TW, Owen N (2008). Too little exercise and too much sitting: inactivity physiology and the need for new recommendations on sedentary behavior. Curr Cardiovasc Risk Rep.

[5] Landi F, Abbatecola AM, Provinciali M, Corsonello A, Bustacchini S, Manigrasso L (2010). Moving against frailty: does physical activity matter?. Biogerontology.

[6] Troiano RP, Berrigan D, Dodd KW, Mâsse LC, Tilert T, McDowell M (2008). Physical activity in the United States measured by accelerometer. Med Sci Sports Exerc.

[7] Clark BK, Sugiyama T, Healy GN, Salmon J, Dunstan DW, Shaw JE (2010). Sociodemographic correlates of prolonged television viewing time in Australian men and women: the AusDiab study. J Phys Act Health.

[8] Barnett I, van Sluijs EMF, Ogilvie D (2012). Physical activity and transitioning to retirement: a systematic review. Am J Prev Med.

[9] Slingerland AS, van Lenthe FJ, Jukema JW, Kamphuis CBM, Looman C, Giskes K (2007). Aging, retirement, and changes in physical activity: prospective cohort findings from the GLOBE Study. Am J Epidemiol.

[10] Barnett I, van Sluijs E, Ogilvie D, Wareham NJ (2014). Changes in household, transport and recreational physical activity and television viewing time across the transition to retirement: longitudinal evidence from the EPIC Norfolk cohort. J Epidemiol Community Health.

[11] Touvier M, Bertrais S, Charreire H, Vergnaud AC, Hercberg S, Oppert JM (2010). Changes in leisure-time physical activity and sedentary behaviour at retirement: a prospective study in middle-aged French subjects. Int J Behav Nutr Phys Act.

[12] Lahti J, Laaksonen M, Lahelma E, Rahkonen O (2011). Changes in leisure-time physical activity after transition to retirement: a follow-up study. Int J Behav Nutr Phys Act.

[13] Menai M, Fezeu L, Charreire H, Kesse-Guyot E, Touvier M, Simon C (2014). Changes in sedentary behaviours and associations with physical activity through retirement: a 6-year longitudinal study. PLoS One.

[14] Chung S, Domino ME, Stearns SC, Popkin BM (2009). Retirement and physical activity: analyses by occupation and wealth. Am J Prev Med.

[15] Van Dyck D, Mertens L, Cardon G, De Cocker K, De Bourdeaudhuij I. Opinions towards physical activity, sedentary behavior and interventions to stimulate active living during early retirement: a qualitative study in recently retired adults. J Aging Phys Act. In press. 10.1123/japa.2015-0295 27759483

[16] Federal Pensions Service Belgium. Available at: http://www.onprvp.fgov.be Accessed 23 Mar 2016.

[17] Craig CL, Marshall AL, Sjöström M, Bauman AE, Booth ML, Ainsworth BE (2003). International Physical Activity Questionnaire: 12-country reliability and validity. Med Sci Sports Exerc.

[18] Salmon J, Owen N, Crawford D, Bauman A, Sallis JF (2003). Physical activity and sedentary behavior: a population-based study of barriers, enjoyment and preference. Health Psychol.

[19] Ware JE, The SCD, MOS (1992). 36-Item Short-Form Health Survey (SF-36): conceptual framework and item selection. Med Care.

[20] Keene ON (1995). The log transformation is special. Stat Med.

[21] Beck F, Gillison F, Standage M (2010). A theoretical investigation of the development of physical activity habits in retirement. Br J Health Psychol.

[22] Barnett I, Guell C, Ogilvie D (2012). The experience of physical activity and the transition to retirement: a systematic review and integrative synthesis of qualitative and quantitative evidence. Int J Behav Nutr Phys Act.

[23] McDonald S, O’Brien N, White M, Sniehotta FF (2015). Changes in physical activity during the retirement transition: a theory-based, qualitative interview study. Int J Behav Nutr Phys Act.

[24] Forsyth A, Oakes MJ, Lee B, Schmitz KH (2009). The built environment, walking, and physical activity: is the environment more important to some people than others?. Transp Res Part D: Transp Environ.

